# Routine laboratory parameters predict intensive care unit admission and hospitalization in patients suffering stab injuries

**DOI:** 10.3389/fimmu.2022.959141

**Published:** 2023-01-05

**Authors:** Tazio Maleitzke, Sijia Zhou, Dario Zocholl, Florian Nima Fleckenstein, David Alexander Back, Julius Maximilian Plewe, Jérôme Weber, Tobias Winkler, Ulrich Stöckle, Serafeim Tsitsilonis, Sven Märdian

**Affiliations:** ^1^ Center for Musculoskeletal Surgery, Charité – Universitätsmedizin Berlin, Corporate Member of Freie Universität Berlin and Humboldt-Universität zu Berlin, Berlin, Germany; ^2^ Julius Wolff Institute, Berlin Institute of Health at Charité – Universitätsmedizin Berlin, Berlin, Germany; ^3^ BIH Charité Clinician Scientist Program, Berlin Institute of Health at Charité – Universitätsmedizin Berlin, BIH Biomedical Innovation Academy, Berlin, Germany; ^4^ Institute of Biometry and Clinical Epidemiology, Charité – Universitätsmedizin Berlin, Corporate Member of Freie Universität Berlin and Humboldt-Universität zu Berlin, Berlin, Germany; ^5^ Department of Diagnostic and Interventional Radiology, Charité – Universitätsmedizin Berlin, Corporate Member of Freie Universität Berlin and Humboldt-Universität zu Berlin, Berlin, Germany; ^6^ Department of Traumatology and Orthopaedics, Septic and Reconstructive Surgery, Bundeswehr Hospital Berlin, Berlin, Germany; ^7^ Department of Surgery, Charité – Universitätsmedizin Berlin, Corporate Member of Freie Universität Berlin and Humboldt-Universität zu Berlin, Berlin, Germany; ^8^ Berlin Institute of Health Center for Regenerative Therapies, Berlin Institute of Health at Charité – Universitätsmedizin Berlin, Berlin, Germany

**Keywords:** trauma, knife, penetrating, blood, serum biochemistry, coagulation, hemostasis, inflammation

## Abstract

**Background:**

Knife crime has increased considerably in recent years in Northern Europe. Affected patients often require immediate surgical care due to traumatic organ injury. Yet, little is known about clinically relevant routine laboratory parameters in stab injury patients and how these are associated with intensive care unit (ICU) admission, hospitalization and number of surgeries.

**Methods:**

We retrospectively analyzed 258 stab injury cases between July 2015 and December 2021 at an urban Level I Trauma Center. Annual and seasonal incidences, injury site, injury mechanism, Injury Severity Score (ISS), and surgical management were evaluated. First, correlations between routine laboratory parameters for hematology, coagulation, and serum biochemistry (peak, and Δ (change from admission to peak within 3 days following admission)) and length of hospital stay, ICU stay, and number of surgeries were assessed using Spearman’s rank correlation coefficients. Second, multivariable Least Absolute Shrinkage and Selection Operator (LASSO) regression analyses were conducted to identify parameters predictive of clinical outcomes. Third, longitudinal developments of routine laboratory parameters were assessed during hospital admission.

**Results:**

In 2021, significantly more stab injuries were recorded compared with previous years and occurred less during winter compared with other seasons. Mean ISS was 8.3 ± 7.3, and ISS was positively correlated with length of hospital and ICU stay (r = 0.5–0.8, p < 0.001). Aspartate transaminase (AST) (Δ) (r = 0.690), peak C-reactive protein (CrP) (r = 0.573), and erythrocyte count (Δ) (r = 0.526) showed the strongest positive correlations for length of ICU stay for penetrating, thoracoabdominal, and organ injuries, respectively. No correlations were observed between routine laboratory parameters and number of surgeries. For patients with penetrating injuries, LASSO-selected predictors of ICU admission included ISS, pH and lactate at admission, and Δ values for activated partial thromboplastin time (aPTT), K^+^, and erythrocyte count. CrP levels on day 3 were significantly higher in patients with penetrating (p = 0.005), thoracoabdominal (p = 0.041), and organ injuries (p < 0.001) compared with those without.

**Conclusion:**

Our data demonstrate an increase in stab injury cases in 2021 and an important link between changes in routine laboratory parameters and ICU admission and hospitalization. Monitoring ISS and changes in AST, CrP, erythrocyte count, pH, lactate, aPTT, and K^+^ may be useful to identify patients at risk and adjust surgical and ICU algorithms early on.

## Introduction

Stab injuries are primarily observed in urban environments and predominantly affect young men between 20 and 30 years of age (1, 2). In a recent retrospective analysis of ~240,000 severely injured patients enrolled in the German Trauma Registry (TraumaRegister DGU^®^), 4,333 stab injuries (1.8%) were recorded between 2009 and 2018 (3).

While overall patient volumes declined in emergency departments (EDs) during the COVID-19 pandemic, incidences of stabbing trauma increased in both Europe (4, 5) and the United States (6–9). Although most stab injuries are not fatal, thoracoabdominal injuries often require surgery and are associated with postsurgical complications and hospital readmissions (10).

Stab injuries result in the disruption of macro (skin) and micro (cell membranes) barriers, causing the release of damage-associated molecular patterns (DAMPs) into the bloodstream, which leads to an activation of the innate immune system (11). Pathogen-associated molecular patterns (PAMPs) from bacteria, viruses, or fungi are also commonly present at the injury site following penetrating trauma. Both DAMPs and PAMPs cause a rapid immune response, including activation of coagulation and complement cascades and reprioritizing of leukocytes (12). In addition, endothelial damage and platelet activation cause a pro-inflammatory cytokine release, which facilitates neutrophil activation and subsequent tissue damage (13). Therefore, stab injuries are likely to affect multiple-organ systems, both primarily though the injury and secondarily through the concomitant cellular and molecular response.

Various serum laboratory parameters are regularly acquired upon arrival of stab injury patients in the ED to monitor health status and organ functions. Blood tests commonly include hematology, coagulation, and serum biochemistry profiles and may be repeated during later stages of admission depending on the patient’s clinical development. How stab injuries alter routine laboratory parameters has not yet been investigated in detail. Knowledge about relevant laboratory parameters to monitor in stab injury patients may however improve assessment and prediction of the clinical course and outcomes early on.

Therefore, this study aimed to describe (i) clinical characteristics of stab injury cases according to the Injury Severity Score (ISS); (ii) explore annual and seasonal differences in monthly stab injury incidences; (iii) evaluate whether peak and Δ (change from admission to peak within 3 days following admission) values of routinely acquired laboratory parameters correlate with clinical outcomes, including length of hospital and ICU stay and number of surgeries performed during admission; and (iv) assess the predictive strength of routinely acquired laboratory parameters for clinical outcomes employing a multivariable regression analysis approach; and (v) compare the longitudinal course of routine laboratory parameters between patients with different injury characteristics over time.

## Materials and methods

### Study population and data extraction

All patients admitted to the ED of a Level I Trauma Center (Berlin, Germany) who sustained one or more stab injuries between July 2015 and December 2021 were included and retrospectively reviewed (retrospective chart review employing a convenience sampling approach (14)). All patients were directly admitted to our center (no transfers from other hospitals).

For all patients, the following parameters were extracted: Injury Severity Score (ISS) ((based on injury patterns present at admission); gender; age; time of admission; health insurance status; mental health condition; alcohol consumption; body temperature at admission; injury mechanism; number of stabs; injured body regions; type of injury, X-ray, ultrasonography, and computed tomography (CT) scan reports (acute pathological findings were quantified); trauma team activation (initiated if patients met Grade of Recommendation B-criteria (GoR-B) defined by DGU^®^ (e.g., penetrating injuries) (15)); performed surgeries (including all acute and elective “in house” surgeries and chest drain insertions *via* mini-thoracotomy, whereas simple superficial wound closures in the ED were not included); massive transfusion of blood products (defined as transfusion of ≥10 red blood cell units within 24 h or more than four red blood cell units within 1 h (16)); red blood cell (RBC); fresh frozen plasma (FFP); and platelet units; length of hospital and ICU stay.

As the study population comprised mono- and polytrauma patients, **Table 1** categorizes patients based on ISS at admission: minor injuries (ISS of 1–8), moderate injuries (ISS of 9–15), severe injuries (ISS of 16–24), and very severe injuries (ISS ≥25) (17).

**Table 1:** Characteristics of patients who suffered stab injuries between July 2015 and December 2021 at a Level I Trauma Center in Berlin, Germany.

ISS		Total	1–8	9–15	16–24	>24	*p* value
n (%)		258 (100)	155 (60.0)	64 (24.8)	28 (10.9)	11 (4.3)	
**Gender, n (%)**							0.918
Male		235 (91.1)	142 (91.6)	58 (90.6)	25 (89.3)	10 (90.9)	
Female		23 (8.9)	13 (8.4)	6 (9.4)	3 (10.7)	1 (9.1)	
**Age, years, median (IQR)**		32 (23, 42)	32 (23, 43)	32 (22, 40)	34 (26, 44)	24 (21, 37)	0.530
**Time of admission, n (%)**							0.215
Daytime (6 a.m.–6 p.m.)		100 (38.8)	53 (34.2)	27 (42.2)	15 (53.6)	5 (45.5)	
Nighttime (6 p.m.–6 a.m.)		158 (61.2)	102 (65.8)	37 (57.8)	13 (46.4)	6 (54.5)	
**Health insurance status, n (%)**							0.217
Insurance cover		231 (89.5)	135 (87.1)	60 (93.8)	27 (96.4)	9 (81.8)	
No insurance cover		27 (10.5)	20 (12.9)	4 (6.2)	1 (3.6)	2 (18.2)	
**Mental health condition, n (%)**							0.227
Reported mental illness		45 (17.4)	21 (13.5)	15 (23.4)	7 (25.0)	2 (18.2)	
No reported mental illness		213 (82.6)	134 (86.5)	49 (76.6)	21 (75.0)	9 (81.8)	
**Alcohol consumption, n (%)**							0.224
Alcohol involved		54 (20.9)	31 (20.0)	11 (17.2)	10 (35.7)	2 (18.2)	
Alcohol not involved		204 (79.1)	124 (80.0)	53 (82.8)	18 (64.3)	9 (81.8)	
**Injury mechanism, n (%)**							0.330
Self-inflicted		48 (18.6)	25 (16.1)	16 (25.0)	4 (14.3)	3 (27.3)	
Assault-related		210 (81.4)	130 (83.9)	48 (75.0)	24 (85.7)	8 (72.7)	
**Injured body region, n (%)**							
Head and neck	With	53 (20.5)	32 (20.6)	12 (18.8)	7 (25.0)	2 (18.2)	0.918
	Without	205 (79.5)	123 (79.4)	52 (81.3)	21 (75.0)	9 (81.8)	
Thorax	With	133 (51.6)	58 (37.4)	46 (71.9)	20 (71.4)	9 (81.8)	< 0.001
	Without	125 (48.4)	97 (62.6)	18 (28.1)	8 (28.6)	2 (18.2)	
Abdomen	With	89 (34.5)	46 (29.7)	23 (35.9)	13 (46.4)	7 (63.6)	0.057
	Without	169 (65.5)	109 (70.3)	41 (64.1)	15 (53.6)	4 (36.4)	
Spine	With	9 (3.5)	3 (1.9)	3 (4.7)	3 (10.7)	0 (0)	0.123
	Without	249 (96.5)	152 (98.1)	61 (95.3)	25 (89.3)	11 (100)	
Limbs	With	108 (41.9)	76 (49.0)	20 (31.3)	10 (35.7)	2 (18.2)	0.024
	Without	150 (58.1)	79 (51.0)	44 (68.8)	18 (64.3)	9 (81.8)	
**Number of involved body regions, n (%)**							0.055
1		161 (62.4)	104 (67.1)	39 (60.9)	12 (42.9)	6 (54.5)	
2–3		92 (35.7)	50 (32.3)	23 (35.9)	15 (53.6)	4 (36.4)	
4–5		5 (1.9)	1 (0.6)	2 (3.1)	1 (3.6)	1 (9.1)	
**Type of injury, n (%)**							< 0.001
Superficial		64 (24.8)	44 (28.4)	13 (20.3)	5 (17.9)	2 (18.2)	
Penetrating		236 (91.5)	134 (86.5)	63 (98.4)	28 (100)	11 (100)	
Thoracoabdominal		170 (65.9)	78 (50.3)	56 (87.5)	25 (89.3)	11 (100)	
Organ		111 (43.0)	23 (14.8)	52 (81.3)	25 (89.3)	11 (100)	
**CT scan, n (%)**							0.002
Performed		223 (86.4)	124 (80.0)	62 (96.9)	27 (96.4)	10 (90.9)	
Not performed		35 (13.6)	31 (20.0)	2 (3.1)	1 (3.6)	1 (9.1)	
**Trauma team activation, n (%)**							0.007
Yes		211 (81.8)	117 (75.5)	60 (93.8)	25 (89.3)	9 (81.8)	
No		47 (18.2)	38 (24.5)	4 (6.2)	3 (10.7)	2 (18.2)	
**Surgery, n (%)**							< 0.001
Yes		183 (70.9)	94 (60.6)	54 (84.4)	24 (85.7)	11 (100)	
No		75 (29.1)	61 (39.4)	10 (15.6)	4 (14.3)	0 (0)	
**Massive transfusion, n (%)**							< 0.001
Yes		11 (4.3)	0 (0)	0 (0)	6 (21.4)	5 (45.5)	
No		247 (95.7)	155 (100)	64 (100)	22 (78.6)	6 (54.5)	
**Blood product, n [median (range)]**							*-*
Red blood cell (RBC) units		25 [6 (1, 46)]	3 [3 (1, 8)]	8 [1 (1, 6)]	8 [11 (2, 14)]	6 [12 (2, 46)]	
Fresh frozen plasma (FFP) units		18 [10 (2, 72)]	2 [5 (3, 6)]	4 [5 (2, 7)]	5 [15 (14, 19)]	7 [10 (3, 72)]	
Platelet units		4 [7 (2, 14)]	0 [0 (0, 0)]	0 [0 (0, 0)]	2 [6 (2, 9)]	2 [9 (4, 14)]	
**Length of stay, days, median (IQR)**							*-*
Length of hospital stay		4 (3, 7)	3 (2, 4)	6 (5, 8)	10 (8, 14)	10 (3, 15)	
Length of ICU stay		0 (0, 2)	0 (0, 0)	2 (0, 3)	3 (2, 7)	3 (0, 11)	

Distribution according to ISS.

According to admission dates, differences in monthly stab injuries between years (2015–2021) and seasons (spring: March–May; summer: June–August; autumn: September–November; winter: December–February) were recorded. As the data set for 2015 only included data for July–December, it was excluded for the assessment of seasonal differences.

Next to demographic data, laboratory values for hematology, coagulation, serum biochemistry, and venous blood gas were recorded. These included hemoglobin, erythrocyte count, leukocyte count, platelet count (all hematology), international normalized ratio (INR), activated partial thromboplastin time (aPTT), fibrinogen (all coagulation), creatinine, urea, total bilirubin, myoglobin, alanine transferase (ALT), aspartate transferase (AST), alkaline phosphatase (ALP), creatine kinase (CK), potassium (K^+^), C-reactive protein (CrP) (all serum biochemistry), and pH and lactate at admission (both venous blood gas).

### Peak and Δ laboratory parameters

To explore clinically relevant changes in routine laboratory parameters in stab injury patients, Δ and peak values were calculated. Δ values express the difference between admission and peak levels within 3 days of admission (Δ = peak level until day 3-admission level) (18).

### Statistical analysis

To test for relationships between groups of different injury severities (according to ISS), chi-square test and Fisher’s exact test were employed to analyze variables in contingency **Table 1**.

The non-parametric Mann–Whitney U test was used to compare continuous or ordinal variables. Due to the asymmetric distribution of laboratory values, the median was reported.

Associations between included laboratory parameters and length of hospital and ICU stay and number of surgeries were evaluated by Spearman’s rank correlation coefficients. Following Mukak’s guide to the appropriate use of correlation coefficients in medical research, correlation coefficients were categorized as negligible (r = 0–0.3), weak (r = 0.3–0.5), moderate (r = 0.5–0.7), high (r = 0.7–0.9), and very high (r = 0.9–1) (19). Correlation analyses were conducted for patient cohorts with (i) penetrating (all injuries where the visceral or deep fascia was perforated), (ii) thoracoabdominal, and (iii) organ injuries.

To further identify clinically meaningful relationships from the multivariate data set (20), a multivariable selection method was employed to identify potentially predictive sets of variables: LASSO [Least Absolute Shrinkage and Selection Operator (21)] analyses were conducted for each patient cohort of (i) penetrating, (ii) thoracoabdominal, and (iii) organ injuries and each outcome of interest. LASSO models have been commonly used for high-dimensional model selection problems, i.e., if there are numerous predictive factors for a limited sample size. LASSO applies a shrinkage parameter *lambda*, which shrinks some coefficients of a regression model to zero, thus selecting only those variables that are useful for prediction. The specific value of *lambda* determines how many non-zero coefficients are considered. Due to this shrinkage, coefficient estimates are biased by design and p-values or confidence intervals do not have reliable statistical properties (22). Therefore, we report trace plots, showing coefficients of each variable for different lambda values. To identify a single model, a suitable value for lambda was determined *via* cross-validation. The identified model was then used to predict admission to ICU, and sensitivity, specificity, and total accuracy of this prediction were assessed as well as the area under the receiver operating characteristic curve (AUC). Since multivariable regression methods require non-missing data on all variables, a subset of patients with most complete data (n = 66) was used and only variables with less than 120 missing values were considered for variable selection.

It should be noted that the models were purely explorative and should be used to identify potentially predictive factors for future research rather than serve as a final predictive model.

Additionally, longitudinal changes of CrP, hemoglobin, erythrocyte, leukocyte, and platelet counts, total bilirubin, ALT, AST, creatinine, urea, CK, INR, and aPTT were recorded for 21 days following admission and the day of discharge.

To compare daily CrP values between patients with different injury characteristics, patient cohorts were compared according to (i) penetrating injuries, (ii) thoracoabdominal injuries, and (iii) organ injuries to those without, as well as in (iv) patients who were admitted to ICU to those who were not. Where less than four data points were available per group, no statistical tests were conducted.

For Spearman correlation, LASSO regression, and longitudinal laboratory parameter analyses, 26 patients were excluded. These included 18 patients who left the hospital against medical advice, five patients who died in hospital due to their injuries, and three patients who were initially transferred to specialized medical departments for further treatment.

A two-tailed p-value <0.05 was considered statistically significant. Data analyses were conducted using SPSS 26.0 (IBM, Chicago, USA) and Prism V.9.0 (GraphPad, California, USA) and R version 4.1.2 [for LASSO analysis: package glmnet (23)].

### Ethics

Prior to study initiation, ethical approval (EA1/082/20) was obtained from the local ethics committee, Ethikkommission Charité – Universitätsmedizin Berlin.

## Results

### Descriptive data of stab injury patients

Between July 2015 and December 2021, 258 stab injuries were identified (23 women, 235 men) with a median age of 32 years (interquartile range (IQR) 23; 42). Most stab injuries occurred between 6 p.m. and 6 a.m. (61.2%) and were due to interpersonal conflicts (81.4%), whereas 18.6% were self-inflicted. In 17.4% of sustained stab injuries, mental illness was recorded, and in 20.9% elevated blood alcohol levels or obvious alcohol intoxication were present.

**Figure 1:**
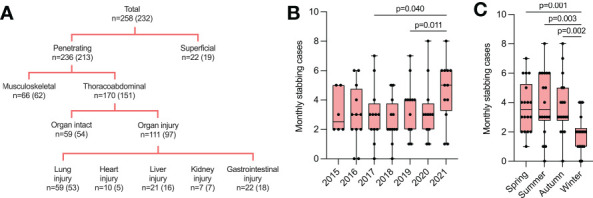
Annual and seasonal differences in monthly stab injuries. **(A)** Characteristics of included stab injuries divided by penetrating vs. superficial, thoracoabdominal vs. musculoskeletal, and organ injuries vs. non-organ injuries. Numbers in brackets show the number of cases included in statistical analysis (for details, please see Materials and Methods). **(B)** Monthly stab injury cases between 2015 and 2021. Individual data points represent the number of stabbing cases per month for indicated years. **(C)** Monthly stab injury cases between 2016 and 2021 divided by seasons. Individual data points represent the number of stabbing cases per month for indicated seasons. Data are presented as median box plots with interquartile range and whiskers from minimum to maximum.

The trauma team was activated in 81.8% of cases, and 70.9% of patients underwent surgery. More than half of the patients (51.6%) sustained a thorax injury, whereas the limbs and abdomen were affected in 41.9% and 34.5% of cases, respectively. Most patients presented with penetrating injuries (91.5%), and 43.0% sustained organ injuries (**Figure 1A**; **Table 1**). The detailed distribution of cases according to ISS is provided in **Table 1**.

### Annual and seasonal differences in monthly stab injuries between 2015 and 2021

We recorded significantly more monthly stab injuries in 2021 compared to 2017 (p = 0.040) and 2018 (p = 0.011) (**Figure 1B**). Most stabbings occurred during spring, summer, and autumn, whereas significantly fewer cases were seen during winter (spring vs. winter, p = 0.001; summer vs. winter, p = 0.003; autumn vs. winter, p = 0.002) (**Figure 1C**).

### Correlations between laboratory parameters and clinical outcomes and predictive variables for ICU admission in penetrating injuries

We next analyzed how injury severity and routine laboratory parameters correlated with length of hospital and ICU stay and number of surgeries in patients with penetrating stab injuries (n = 213). The median time from admission to first blood samples was 35 min (IQR 28–41 min) for blood hematology, biochemistry, and coagulation samples and 10 min (IQR 10–18 min) for venous blood gas samples.

**Figure 2:**
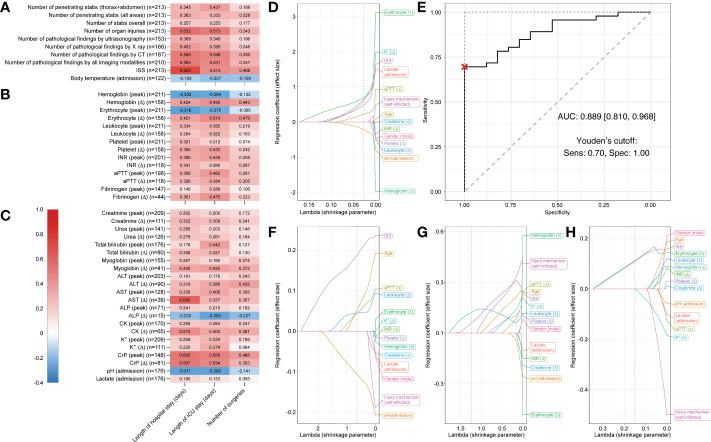
Correlation matrix showing Spearman’s rank correlation coefficients and Least Absolute Shrinkage and Selection Operator (LASSO) plots showing regression coefficients for patients who suffered penetrating stab injuries. Correlation strength is indicated by color scale on the left. Correlations between three different outcome parameters (length of hospital stay, length of ICU stay, and number of surgeries) and **(A)** injury specifics, **(B)** routine hematology and coagulation, and **(C)** serum biochemistry and venous blood gas laboratory parameters for penetrating injuries (n = 213). Multivariable LASSO trace plots indicate potentially predictive sets of variables for **(D)** ICU admission (yes/no). **(E)** Receiver operating characteristic (ROC) curve for the LASSO model of ICU admission (yes/no). Youden’s cutoff indicates the optimal cutoff based on sensitivity (Sens.) and specificity (Spec). **(F)** Further LASSO trace plots for length of hospital stay, **(G)** length of ICU stay, and **(H)** number surgeries. Grey dotted line displays optimal lambda value calculated *via* cross-validation. AUC, area under the curve.

Number of organ injuries, number of pathological CT findings, number of pathological findings in all imaging modalities combined, and ISS were all moderately to highly positively correlated with length of hospital and ICU stay (r = 0.513–0.805, p < 0.001 for all) (**Figure 2A**).

Further, AST (Δ), CK (Δ), peak CrP, and CrP (Δ) (**Figure 2C**) were all moderately positively correlated with length of hospital stay (r = 0.510–0.690) and erythrocyte count (Δ) (**Figure 2B**), peak CrP, and CrP (Δ) (**Figure 2C**) were also moderately positively correlated with length of ICU stay (r = 0.510–0.558).

The strongest positive correlations were observed between ISS and hospital stay (r = 0.805) and AST (Δ) and ICU stay (r = 0.690). For all reported r-values >0.3, p-values were <0.01. Negligible to weak correlations were observed between all assessed parameters and number of surgeries.

For admission to ICU (yes/no), LASSO regression analyses selected the following predictive variables when shrinkage parameter lambda was set to 0.027 (determined *via* cross-validation): pH (admission) (coefficient = -0.213), aPTT (Δ) (coefficient = 0.219), ISS (coefficient = 0.479), lactate (admission) (coefficient = 0.594), K^+^ (Δ) (coefficient = 0.657), and erythrocyte count (Δ) (coefficient = 0.696) (**Figure 2D**).

With these predictive variables, a binary logistic regression model predicting ICU admission had a sensitivity of 73.9% (95% confidence interval [58.7%, 95.7%]), a specificity of 100% [76.5%, 100.0%], and a total accuracy of 81% [69.8%, 92.1%]. The AUC was 0.889 [0.810, 0968] (**Figure 2E**). Thus, prediction by this model was statistically significantly better than random. In the trace plot, it can be seen that other parameters became dominant with smaller values of lambda. For instance, hemoglobin (Δ) was the largest negative coefficient for lambda values close to zero (**Figure 2D**).

For length of hospital stay the variable ISS, for length of ICU stay hemoglobin (Δ), and for number of surgeries male gender were the largest coefficients for lambda values close to zero (**Figures 2F–H**). Details on LASSO-selected variables and their coefficients can be found in **Table 2**.

**Table 2:** LASSO-selected predictive variables with corresponding coefficients (n = 66).

	Penetrating injuries				Thoracoabdominal injuries				Organ injuries			
	ICU admission (yes/no)	Length of hospital stay	Length of ICU stay	Number of surgeries	ICU admission (yes/no)	Length of hospital stay	Length of ICU stay	Number of surgeries	ICU admission (yes/no)	Length of hospital stay	Length of ICU stay	Number of surgeries
Lambda (shrinkage parameter)	0.027	1.231	0.647	0.150	0.034	0.311	0.329	0.150	0.048	1.497	0.987	0.212
Selected variables (effect size)												
Gender (male)	–	–	–	–	0.478	–	–	–	0.454	–	–	–
Age	–	–	0.052	–	–	–	–	–	–	–	0.080	–
Injury mechanism (self-inflicted)	–	–	–	–	–	–	0.350	–	–	–	0.282	–
ISS	0.479	0.061	0.188	0.109	0.589	0.277	0.410	0.050	–	0.061	–	0.153
Hemoglobin (Δ)	–	–	–	–	–	–	0.003	0.008	–	–	–	
Erythrocyte count (Δ)	0.696	–	–	0.112	0.564	0.015	0.019	0.153	0.911	–	–	0.069
Leukocyte count (Δ)	–	0.123	0.045	–	–	–	–	0.007	0.088	0.017	0.181	0.003
aPTT (Δ)	0.219	–	0.044	–	0.175	0.026	–	–	–	–	–	–
Creatinine (Δ)	–	–	–	–	–	–	–	–	0.643	–	0.201	–
K^+^ (Δ)	0.657	–	–	–	0.592	–	0.096	–	0.502	–	–	–
pH (admission)	-0.213	–	-0.106	–	-0.079	-0.056	-0.097	–	-0.064	-0.033	–	–
Lactate (admission)	0.594	–	–	–	0.562	–	–	–	–	–	–	–

### Correlations between laboratory parameters and clinical outcomes and predictive variables for ICU admission in thoracoabdominal injuries

Next, we assessed patients who suffered penetrating injuries that affected thoracoabdominal cavities (n = 151).

**Figure 3:**
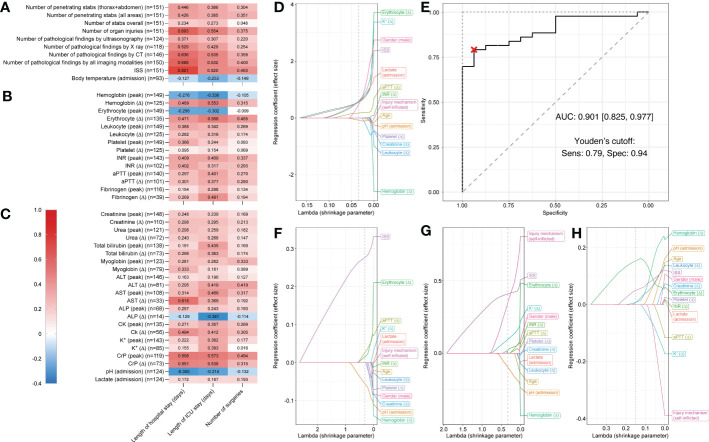
Correlation matrix showing Spearman’s rank correlation coefficients and Least Absolute Shrinkage and Selection Operator (LASSO) plots showing regression coefficients for patients who suffered thoracoabdominal stab injuries. Correlation strength is indicated by color scale on the left. Correlations between three different outcome parameters (length of hospital stay, length of ICU stay, and number of surgeries) and **(A)** injury specifics, **(B)** routine hematology and coagulation, and **(C)** serum biochemistry and venous blood gas laboratory parameters for penetrating injuries (n = 151). Multivariable LASSO trace plots indicate potentially predictive sets of variables for **(D)** ICU admission (yes/no). **(E)** Receiver operating characteristic (ROC) curve for the LASSO model of ICU admission (yes/no). Youden’s cutoff indicates the optimal cutoff based on sensitivity (Sens.) and specificity (Spec). **(F)** Further LASSO trace plots for length of hospital stay, **(G)** length of ICU stay, and **(H)** number surgeries. Grey dotted line displays optimal lambda value calculated *via* cross-validation. AUC, area under the curve.

Number of organ injuries, number of pathological X-ray findings, number of pathological CT findings, and number of pathological findings in all imaging modalities combined, ISS (**Figure 3A**), AST (Δ), peak CrP, and CrP (Δ) (**Figure 3C**) were moderately to highly positively correlated with length of hospital stay (r = 0.529–0.821). Similarly, number of organ injuries, number of pathological CT findings, and number of pathological findings in all imaging modalities combined, ISS (**Figure 3A**), hemoglobin (Δ), erythrocyte count (Δ) (**Figure 3B**), peak CrP, and CrP (Δ) (**Figure 3C**) showed moderate to high positive correlations with length of ICU stay (r = 0.520–0.821). The strongest positive correlations were again observed between ISS and hospital stay (r = 0.821) and between peak CrP and ICU stay (r = 0.573). For all reported r-values > 0.3, p-values were <0.01 and only negligible to weak correlations were observed between all assessed parameters and number of surgeries.

For admission to ICU (yes/no), LASSO regression analyses selected variables pH (admission) (coefficient = -0.079), aPPT (Δ) (coefficient 0.175), male gender (coefficient = 0.478), lactate (coefficient = 0.562), erythrocyte count (Δ) (coefficient = 0.564), ISS (coefficient = 0.589), and K^+^ (Δ) (coefficient = 0.592) when shrinkage parameter lambda was set to 0.034 (determined *via* cross-validation) (**Figure 3D**).

The binary logistic regression model predicting ICU admission had a sensitivity of 79.1% (95% confidence interval [62.8%, 93%]), a specificity of 100% [81.2%, 100.0%], and a total accuracy of 84.7% [72.9%, 93.2%]. The AUC was 0.901 [0.825, 0.977] (**Figure 3E**). Thus, prediction by this model was statistically significantly better than random (**Figure 3D**).

For length of hospital stay the variable ISS, for length of ICU stay self-inflicted injuries, and for number of surgeries hemoglobin (Δ) were the largest coefficients for lambda values close to zero (**Figures 3F–H**). Details on LASSO-selected variables and their coefficients can be found in **Table 2**.

### Correlations between laboratory parameters and clinical outcomes and predictive variables for ICU admission in organ injuries

**Figure 4:**
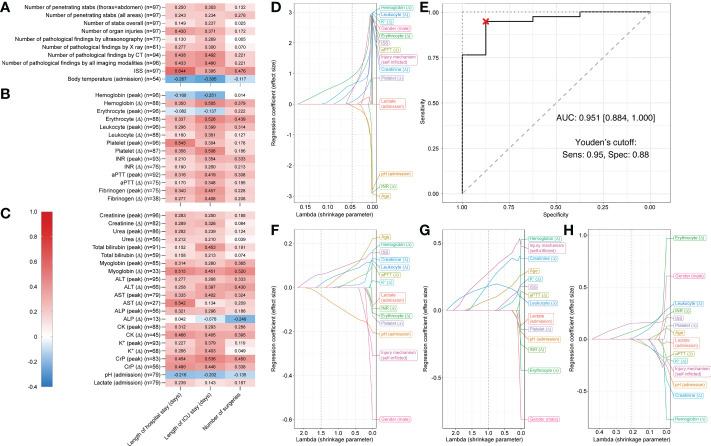
Correlation matrix showing Spearman’s rank correlation coefficients and Least Absolute Shrinkage and Selection Operator (LASSO) plots showing regression coefficients for patients who suffered organ injuries. Correlations between three different outcome parameters (length of hospital stay, length of ICU stay, and number of surgeries) and **(A)** injury specifics, **(B)** routine hematology and coagulation, and **(C)** serum biochemistry and venous blood gas laboratory parameters for penetrating injuries (n = 97). Multivariable LASSO trace plots indicate potentially predictive sets of variables for **(D)** ICU admission (yes/no). **(E)** Receiver operating characteristic (ROC) curve for the LASSO model of ICU admission (yes/no). Youden’s cutoff indicates the optimal cutoff based on sensitivity (Sens.) and specificity (Spec). **(F)** Further LASSO trace plots for length of hospital stay, **(G)** length of ICU stay, and **(H)** number surgeries. Gray dotted line displays optimal lambda value calculated *via* cross-validation. AUC, area under the curve.

When only analyzing patients who suffered organ injuries (n = 97), length of hospital and ICU stay showed moderate positive correlations with ISS (**Figure 4A**), hemoglobin (Δ), erythrocyte count (Δ), peak platelet count, platelet count (Δ) (**Figure 4B**), myoglobin (Δ), AST (Δ), and peak CrP (r = 0.505–0.644) (**Figure 4C**). The strongest positive correlation was again observed between ISS and hospital stay (r = 0.644) and between erythrocyte count (Δ) and ICU stay (r = 0.526). For all reported r-values > 0.3, p-values were <0.01. Again, only negligible to weak correlations were observed between all assessed parameters and number of surgeries.

For admission to ICU (yes/no), LASSO regression analyses selected variables pH (admission) (coefficient = -0.064), leukocyte count (Δ) (coefficient = 0.088), male gender (coefficient = 0.454), K^+^ (Δ) (coefficient = 0.502), creatinine (Δ) (coefficient = 0.643), and erythrocyte count (Δ) (coefficient = 0.911), when shrinkage parameter lambda was set to 0.048 (determined *via* cross-validation) (**Figure 4D**).

The binary logistic regression model predicting ICU admission showed a sensitivity of 92.1% (95% confidence interval [71.1%, 100%]), a specificity of 100% [75.01%, 100.0%], and a total accuracy of 93.5% [76.1%, 100%]. The AUC was 0.941 [0.884, 1.000] (**Figure 4E**). Thus, prediction by this model was statistically significantly better than random. In the trace plot, it can be seen that other parameters became dominant with smaller values of lambda. For instance, hemoglobin (Δ) was the largest coefficient for lambda values close to zero (**Figure 4D**).

For length of hospital stay age, for length of ICU stay hemoglobin (Δ), and for number of surgeries erythrocyte count (Δ) were the largest coefficients for lambda values close to zero (**Figures 4F–H**). Details on LASSO-selected variables and their coefficients can be found in **Table 2**.

### Longitudinal development of daily CrP levels in penetrating, thoracoabdominal, and organ injury cases and in patients admitted to ICU

As a next step, we assessed the longitudinal development of CrP values following stab injuries over time in different patient cohorts.

**Figure 5:**
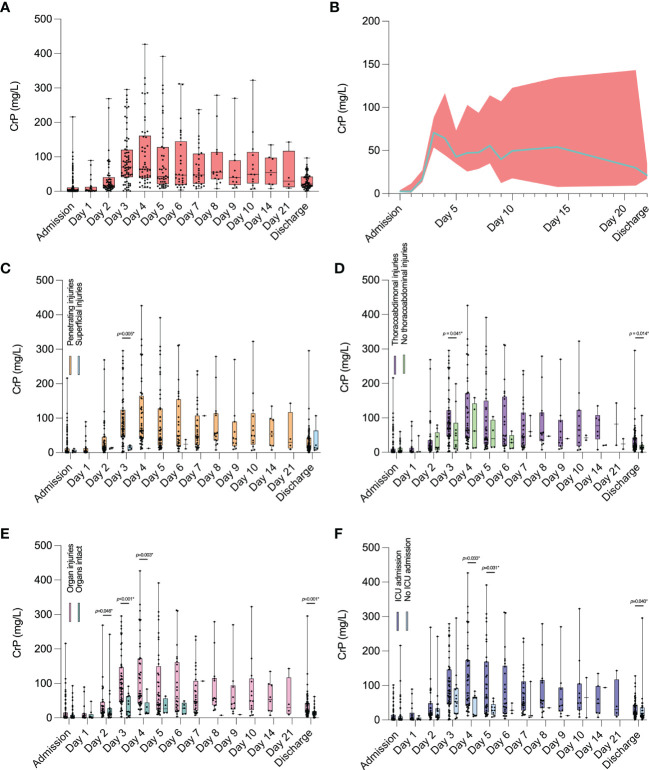
Longitudinal development of daily CrP levels in patients who suffered stab injuries. **(A, B)** Values for all patients who suffered stab injuries. **(C)** Comparison of CrP values between penetrating and superficial injuries during hospitalization. **(D)** Comparison of CrP values between patients with and without thoracoabdominal injuries, **(E)** between patients with and without organ injuries, and **(F)** between patients who were admitted to ICU and those who were not. Physiological CrP concentration: <5 mg/l. Data are presented as median box plots with interquartile range and whiskers from minimum to maximum and in **(B)** as median line with 95% confidence intervals displayed as error bands. CrP, C-reactive peptide.

When assessing all patients (superficial and penetrating injuries combined; patients excluded for correlation and LASSO regression analyses were also excluded here), an increase in CrP levels was observed between admission and day 4. Values then peaked between days 4 and 6 before slowly decreasing again (**Figures 5A, B**).

When comparing patients with penetrating injuries to those with superficial injuries, CrP levels were significantly higher in the penetrating group on day 3 following admission (p = 0.005) (**Figure 5C**).

Comparing patients with thoracoabdominal injuries to those without, CrP levels were again significantly higher on day 3 following admission (p = 0.041) and on the day of discharge (p = 0.014) (**Figure 5D**).

CrP values were also significantly higher on days 2 (p = 0.046), 3 (p < 0.001), and 4 (p = 0.003) following admission, as well as on the day of discharge (p = 0.001) in patients with organ injuries compared to those without (**Figure 5E**).

Finally, in patients admitted to ICU, CrP levels were significantly higher on days 4 (p = 0.033) and 5 (p=0.031) following admission and on the day of discharge (p = 0.040) compared to patients who were not admitted to ICU (**Figure 5F**).

### Longitudinal development of daily laboratory parameters in stab injury patients

To illustrate the longitudinal development of routine laboratory parameters in stab injury patients, we provided an overview for the most relevant parameters, obtained from all patients (superficial and penetrating injuries combined; patients excluded for correlation and LASSO regression analyses were also excluded here).

The longitudinal development of hemoglobin and erythrocyte count over time followed a reversed hockey stick pattern, with physiologically high values at admission, followed by a marked decrease on days 1 and 2 and a stable plateau phase until day 21 (**Supplementary Figure S1A**). Both hemoglobin and erythrocyte count levels were lower on the day of discharge compared to admission.

Leukocyte and platelet counts followed an S-curve over time (**Supplementary Figure S1B**). Compared to admission, leukocyte levels increased on day 1, then decreased until day 5, and increased again until day 10, before decreasing again until day 21. Platelet levels decreased on day 1 compared to admission but followed a similar pattern to leukocyte counts over time (**Supplementary Figure S1B**).

Longitudinal development plots for total bilirubin, ALT, AST, creatinine, urea, CK, K^+^, hemoglobin, erythrocyte, leukocyte and platelet counts, INR, and aPTT can be found in **Supplementary Figures S1C–F**.

## Discussion

In this retrospective study, annual and seasonal differences in stab injury incidences were assessed at an urban Level I Trauma Center. We further investigated how routine laboratory parameters in stab injury patients correlated with and predicted ICU admission, length of hospital and ICU stay, and number of surgeries performed during admission.

We found that number of organ injuries, the number of acute pathological findings in several imaging modalities, ISS, and Δ values for AST, CK, and CrP correlated moderately to highly with hospitalization length. Only negligible to weak correlations were observed between assessed parameters and number of surgeries. Further, we identified ISS, pH, and lactate at admission, and Δ values for aPPT, K^+^, and erythrocyte count as predictors for ICU admission in patients suffering penetrating stab injuries.

ISS and new ISS (NISS) are widely used to predict mortality and morbidity in patients suffering multiple trauma, and both scores correlate with length of hospital stay (24) and can predict ICU admission (25). In this study, we confirmed that ISS may also predict ICU admission and length of hospital stay in patients suffering stab injuries as a higher ISS was associated with longer hospitalization.

The study period in the present study covered more than 20 months during the COVID-19 pandemic (2020/2021), a period that was, to our knowledge, previously not evaluated regarding stab injuries.

Several studies assessed stab injury incidences during the COVID-19 pandemic, and most found a rise in penetrating injuries during the first months of 2020 (4–9), but not all (26, 27). Conversely, it was reported that during the first months of the COVID-19 pandemic, crime rates, including assault, decreased globally (28).

We found that stab injuries significantly increased in 2021, i.e., in the later phase of the pandemic and after most COVID-19-related measures had been lifted in Germany. This is congruent with studies demonstrating that several psychological effects occurring after crises (posttraumatic stress disorder, anxiety, fear, and anger) may only be observed with a certain delay (29, 30). A study from Mexico showed that assault and battery numbers decreased in the beginning of the COVID-19 pandemic but increased again when national lockdowns were lifted (31). Congruently, data from the UK showed a return of penetrating injury numbers to pre-pandemic levels, following an initial decline during the first months of the COVID-19 pandemic (27). In line with our findings, observations from natural catastrophes showed a link between length of exposure to natural disasters and increased violence (32, 33), which may explain why significantly more stab injuries were seen during the later phases of the COVID-19 pandemic, in 2021.

We further found that stab injuries occurred less often during winter than during other seasons. This is concordant with data from Ho et al., who reported a positive association between warm weather and penetrating trauma (34). A study from the UK demonstrated that stabbings in young adults mainly occurred after midnight (35). In accordance, we showed that over 60% of stab injury patients were admitted between 6 p.m. and 6 a.m.

From a clinical and surgical perspective, changes in blood composition are of particular interest in patients suffering penetrating organ injuries. Platelet dysfunction following trauma has previously been linked to increased mortality (36, 37), however, to the best of our knowledge, stab injury-associated thrombocytopenia has not yet been investigated.

In the current study, we found Δ platelet values, which take admission values into account, to be positively correlated with length of ICU stay for patients who suffered organ injuries.

Due to blood loss and dilution following intravenous fluid administration, platelet counts may be reduced in patients suffering stab injuries. In severely injured patients, early administration of platelets is therefore advised, as it was shown to improve hemostasis and reduce mortality (38).

For patients with penetrating and thoracoabdominal injuries, aPTT (Δ) was identified as a predictor for ICU admission. While previous research showed that among other coagulation parameters, aPTT increased significantly during the first hours following trauma (39, 40), we could show that increased aPTT (Δ) values may also predict ICU admission in stab injury patients.

These data underline the importance of monitoring changes in platelet levels and coagulation parameters at and during admission to initiate and adjust early interventions and to avoid prolonged hospitalization.

The acute-phase protein CrP rises in response to trauma and tissue injury. Both penetrating injuries and surgeries disrupt skin barriers and cause an instant and delayed immune response (12). While admission CrP values were generally within the physiological range, we saw that peak CrP values correlated with length of hospital (r = 0.605) and ICU stay (r = 0.558) in patients suffering penetrating stab injuries. Correlations were similar for Δ values.

In penetrating trauma, prophylactic antibiotic treatment is commonly employed to prevent and treat infectious complications, and strong evidence exists for prophylactic antibiotic treatment for chest wounds that require tube thoracostomy (41).

During the first days of admission, we observed that patients with penetrating stab injuries showed higher CrP values than those without. This also applied to patients with thoracoabdominal injuries, organ injuries, and patients who were admitted to ICU. Similarly, CrP values at discharge were still significantly higher in patients with thoracoabdominal injuries, organ injuries, and ICU admission than in those without.

High CrP concentrations (values higher than 75 mg/l at ICU discharge (42–44) and hospital discharge (45)) were reported as predictors of poor clinical outcomes and increased readmission rates in critically ill patients including multiple trauma, burns, and open heart surgery patients. However, conflicting evidence suggests that CrP values at ICU discharge are unrelated to readmission rates or death (46, 47). Therefore, determining the right time point to discharge a patient requires thorough and repeated clinical assessments in addition to CrP monitoring and must be decided individually.

However, if stab injury patients fall into one of the aforementioned categories (penetrating, thoracoabdominal, organ injuries, or ICU admission), inflammation and sepsis markers including CrP and procalcitonin shall be monitored in addition to the patient’s clinical state (48, 49). Further, early admission of prophylactic antibiotics should be considered to potentially reduce hospitalization time.

Tissue damage or hemorrhagic shock commonly cause hyperkalemia in trauma patients (50, 51). Importantly, hyperkalemia is associated with acute kidney injury following severe trauma (52). In our study population, we were able to identify K^+^ (Δ) as a predictor of ICU admission in all assessed patient groups (penetrating, thoracoabdominal, and organ injuries).

Hemorrhagic shock and infectious complications can further trigger and exacerbate trauma-related acute kidney injury (53), and elevated CK levels, mainly due to muscle injury, compromise kidney function additionally (54). In line with these findings, we identified creatinine (Δ) as a predictor of ICU admission in patients suffering organ injuries and CK (Δ) correlated with length of hospital stay in patients suffering penetrating stab injuries.

Data from the ongoing UK-based *Activation of Coagulation and Inflammation in Trauma* (ACIT) study showed that trauma-induced acute kidney injury occurred in more than 10% of multiple trauma patients (n = 1,410) (55). Therefore, monitoring and treating acute kidney injury by avoiding fluid volume deficit, nephrotoxic agents and by facilitating blood pressure support may decrease the risk of complications and mortality (53, 56). Fluid overload, which may worsen acute kidney injury, shall be avoided simultaneously (57, 58).

Prehospital lactate levels were previously shown to predict resuscitative care in trauma patients and may be used to aid triage of normotensive trauma patients (59). High lactate and low pH additionally predicted 72 h mortality in multiple trauma patients (60). Coherently, we identified increased lactate and low pH levels at admission as predictors of ICU admission in penetrating and thoracoabdominal injury patients following stabbing trauma.

Last, we identified Δ values of AST to positively correlate with length of hospital stay in patients with penetrating, thoracoabdominal and organ injuries. Previously, liver transaminase levels were shown to predict liver injury following blunt and penetrating trauma (61, 62).

Our data indicate that even in the absence of organ injuries, monitoring liver and kidney function and adjusting treatment algorithms early on may help to avoid prolonged hospitalization in stab injury patients.

This study has several limitations. First, this is a retrospective single-center study conducted at an urban Level I Trauma Center, which only allows limited generalization of the findings, especially regarding other geographical regions and countries. These results may also not translate to rural or remote areas where transfers to Level I Trauma Centers commonly cause delayed admissions. These findings should thus be corroborated by further prospective studies from different regions.

Second, the presence of non-medical factors that may have affected ICU admission and discharge could not be taken into consideration in this study (e.g., bed availability due to COVID-19 patients).

Third, due to the observational character of this study, we identified correlations and predictors but can only speculate about causality. Due to the exploratory nature of this study, confounding factors were not included in the analyses and study design.

In summary, we provide solid evidence that routine laboratory parameters have a predictive strength for outcome parameters in patients who suffer stab injuries. Monitoring specific laboratory parameters in stab injury patients may help to identify risk groups and adjust treatment algorithms early on.

## Data availability statement

The original contributions presented in the study are included in the article/**Supplementary Material**. Further inquiries can be directed to the corresponding author.

## Ethics statement

The studies involving human participants were reviewed and approved by Ethikkommission Charité – Universitätsmedizin Berlin. Written informed consent from the participants’ legal guardian/next of kin was not required to participate in this study in accordance with the national legislation and the institutional requirements.

## Author contributions

TM and SM designed and conceived the study. SZ, DZ, TM, FNF, JMP, and JW provided, selected, assembled, analyzed, and interpreted data. Data curation and project administration by DAB, TW, US, and ST. SM provided project supervision and resources. TM drafted the original manuscript. All authors critically reviewed and edited the final manuscript, agreed to be accountable for all aspects of the work and have read and confirmed that they meet ICMJE criteria for authorship.

## Supplementary material

Supplementary Figure S1 Longitudinal development of daily laboratory values in stab injury patients. **(A)** hemoglobin/erythrocyte count, **(B)** leukocyte count/platelet count, **(C)** total bilirubin/ALT/AST, **(D)** creatinine/urea, **(E)** CK/K+, and (F) INR/aPTT. Data are presented as median box plots with interquartile range and whiskers from minimum to maximum. ALT, alanine transaminase; aPTT, activated partial thromboplastin time; AST, aspartate transaminase; CK, creatine kinase; INR, International Normalized Ratio.
